# Performance of aseptic technique during neuraxial analgesia for labor before and after the publication of international guidelines on aseptic technique

**DOI:** 10.1186/2045-4015-3-9

**Published:** 2014-03-25

**Authors:** Alex Ioscovich, Elyad M Davidson, Sharon Orbach-Zinger, Zvia Rudich, Simon Ivry, Laura J Rosen, Alexander Avidan, Yehuda Ginosar

**Affiliations:** 1 Department of Anesthesiology, Shaare Zedek Medical Center, Jerusalem, Israel; 2 Department of Anesthesiology and Critical Care Medicine, Hadassah Hebrew University Medical Center, POB 12000, Jerusalem 91120, Israel; 3 Department of Anesthesiology, Rabin Medical Center, Petach Tikva, Israel; 4 Department of Anesthesiology, Soroka Hospital, Ben Gurion University, Beer Sheva, Israel; 5 Department of Anesthesiology, Western Galilee Hospital, Nahariyah, Israel; 6 Department of Health Promotion, School of Public Health, Sackler Faculty of Medicine, Tel Aviv University, Ramat Aviv, Israel

## Abstract

**Background:**

Aseptic technique and handwashing have been shown to be important factors in perioperative bacterial transmission, however compliance often remains low despite guidelines and educational programs. Infectious complications of neuraxial (epidural and spinal) anesthesia are severe but fortunately rare. We conducted a survey to assess aseptic technique practices for neuraxial anesthesia in Israel before and after publication of international guidelines (which focused on handwashing, jewelry/watch removal and the wearing of a mask and cap).

**Methods:**

The sampling frame was the general anesthesiology workforce in hospitals selected from each of the four medical faculties in Israel. Data was collected anonymously over one week in each hospital in two periods: April 2006 and September 2009. Most anesthesiologists received the questionnaires at departmental staff meetings and filled them out during these meetings; additionally, a local investigator approached anesthesiologists not present at these staff meetings individually. Primary endpoint questions were: handwashing, removal of wristwatch/jewelry, wearing mask, wearing hat/cap, wearing sterile gown; answering options were: "always", "usually", "rarely" or "never". Primary endpoint for analysis: respondents who *both* always wash their hands *and* always wear a mask ("handwash-mask composite") - "always" *versus* "any other response". We used logistic regression to perform the analysis. Time (2006, 2009) and hospital were included in the analysis as fixed effects.

**Results:**

135/160 (in 2006) and 127/164 (in 2009) anesthesiologists responded to the surveys; response rate 84% and 77% respectively. Respondents constituted 23% of the national anesthesiologist workforce. The main outcome "handwash-mask composite" was significantly increased after guideline publication (33% vs 58%; p = 0.0003). In addition, significant increases were seen for handwashing (37% vs 63%; p = 0.0004), wearing of mask (61% vs 78%; p < 0.0001), hat/cap (53% vs 76%; p = 0.0011) and wearing sterile gown (32% vs 51%; p < 0.0001). An apparent improvement in aseptic technique from 2006 to 2009 is noted across all hospitals and all physician groups.

**Conclusion:**

Self-reported aseptic technique by Israeli anesthesiologists improved in the survey conducted after the publication of international guidelines. Although the before-after study design cannot prove a cause-effect relationship, it does show an association between the publication of international guidelines and significant improvement in self-reported aseptic technique.

## Background

The past decade has been marked by an increased international awareness of the potential infectious complications of neuraxial (epidural and spinal) anesthesia and the need to avoid iatrogenic patient injury. Based on data from published case series [1-4], retrospective cohort studies [5-8], closed claim studies [9,10], national surveys of complications [11-13] and systematic reviews [14-16], the reported incidence of major iatrogenic infectious complications is rare. Meningitis is reported to occur with an incidence between 0.2:10,000 [11] to 1.8:10,000 [13]; epidural abscess is typically reported at approximately 1:100,000 [10], although it has been reported to occur as frequently as 1;1,930 [12] or even 1:800 [2]. Thus, due to the rarity of major infectious complications, accurate assessments of incidence and risk factors are difficult to quantify. In contrast to the rarity of major infectious complications, the reported incidence of local infection after epidural placement is as high as 4.3% [3] and even 12% in a critical care setting [17]. The reported colonization rates are even higher and have been reported as 29% [18] and even as high as 35% [19].

Anesthesiologists as a group have unfortunately acquired some notoriety for non-adherence to basic aseptic technique, particularly for poor standards of handwashing routines [20-22]. This is exacerbated by the physical contact of anesthesiologists with large numbers of surgical patients in the post-anesthesia care unit (PACU) and high-risk infected patients in the intensive care unit (ICU). Hand contamination in anesthesiologists has been shown to be an important factor in intraoperative bacterial transmission [23]. The co-existence of high exposure to infectious agents and poor handwashing routines is a potential concern for the provision of neuraxial anesthesia in healthy laboring women or elective surgical patients. In July 2006, the American Society of Regional Anesthesia (ASRA) published guidelines for aseptic technique for neuraxial anesthesia [24-26]. Important recommendations of these guidelines included the use of a facemask when performing neuraxial analgesia and routine handwashing (including the removal of rings, jewelry and wrist watches).

Studies from other areas of health care have questioned whether guidelines alone have been effective vehicles for change [27]. Furthermore, although compliance with guidelines has been observed to increase following extensive teaching programs, compliance often remains low. One study reported 40% non-adherence to perioperative safety checks despite extensive crew resource management training [28], and another reported 46% non-adherence to guidelines for management of severe hemorrhage after oral anti-coagulant therapy despite a focused lecture program [29]. In practice, change typically requires a comprehensive interaction of both guidelines and educational programs, as part of broader health care policy decisions at local and national levels.

Prior to the impending publication of the ASRA guidelines in 2006 [24-26], we conducted a survey of anesthesiologists in Israel to assess the aseptic technique practiced by Israeli anesthesiologists for neuraxial analgesia for labor. This survey was used as baseline data to assess the impact of these guidelines as an intervention; a follow up survey was performed in 2009. The ASRA guidelines were followed by guidelines developed by the Israel Society of Anesthesiologists (2009) and a practice advisory by the American Society of Anesthesiologists (2010) [30]; both of which were published after the conclusion of our follow-up survey. The national and international guidelines together with their date of publication appear in the Appendix.

## Methods

The population chosen for this study was the anesthesiology departments of the Western Galilee Hospital, Nahariyah; Rabin Medical Center (Campus Beilinson), Petach Tikva; Hadassah Hebrew University Medical Center, Ein Karem; Shaare Zedek Medical Center, both in Jerusalem and Soroka Hospital, Beersheva. All the participating hospitals were chosen as they have relatively high volume maternity and anesthesia services, all have residency programs and all have a service based on a mix of attendings (consultant-status), residents and "non-residents/non-attendings" or NRNA's (physicians who have not passed their board exams but who continue to practice anesthesia under supervision after the expiration of their residency period). While other hospitals also met these criteria, we aimed to base this study on large, representative hospitals affiliated to each of the four medical faculties in Israel at the time of the study (Haifa -Technion, Tel Aviv University, Hebrew University of Jerusalem and Ben Gurion University of the Negev, Beersheva).

The numbers of anesthesiologists in the sampling frame was determined by the number of practicing anesthesiologists at work in each hospital. We assessed general anesthesiologists and did not restrict this survey to anesthesiologists with a predominantly obstetric practice; however anesthesiologists who never provide clinical service to the labor ward (e.g. full time intensive care or pre-operative clinic physicians) were excluded. Questionnaires were distributed by hand to all anesthesiologists in this sampling frame. Most anesthesiologists received the questionnaires at departmental staff meetings and filled them out during these meetings; additionally, a local investigator approached anesthesiologists not present at these staff meetings individually.

The time frame for data collection was over one week in each specific hospital. The first survey was conducted between April to May 2006, the second survey was conducted between September to October 2009.

Questionnaires were filled anonymously and collected immediately. They were placed in an opaque envelope and were not read by the collecting investigator. Individual hospitals were numbered but not identified, so that anonymous hospital data was analyzed statistically.

The questionnaire consisted of three basic elements: 1) demographic data, 2) primary and secondary endpoints (self-reported compliance with aseptic technique for neuraxial analgesia in labor) and 3) other endpoints for descriptive analysis.

Demographic data for the respondents were: age, gender, professional status (attending, resident or NRNA), and number of years in anesthesia practice. Demographic data for the participating hospitals were: number of deliveries per year, epidural rate, and cesarean delivery rate.

Primary and secondary endpoint questions on the self-reported aseptic technique for neuraxial analgesia in labor: handwashing before neuraxial analgesia, removal of wristwatch or jewelry, wearing a face mask, use of a hat/cap and use of a sterile gown. For all of the above, the option for answering was an ordinal series: "always", "usually", "rarely" or "never".

Other queries for descriptive analysis only were as follows: whether they used reusable or disposable drapes, how they washed their hands (soap, alcohol-based antiseptic solution, surgeon's scrub protocol, don't wash) and what solution they used to prep the patient's back. They were asked what aseptic precautions (e.g. face mask, cap) were expected of people in the room (midwife/nurse and patient/family). They were asked whether they were aware of local written protocols for aseptic technique for neuraxial analgesia in their hospital. They were asked if their personal standards of aseptic technique for neuraxial analgesia were adequate in their judgement and whether they were comparable to those of their colleagues. Finally, anesthesiologists were also asked if they had ever had a serious infectious complication (epidural abscess or meningitis) in a patient where they had personally performed the block.

### Statistical analysis

In the demographic data presentation, continuous data (anesthesiologists' age and experience) were presented as median and interquartile range and were compared between groups (2006, 2009) using one-way ANOVA.

Our primary question was whether hygiene levels were similar before and after release of the guidelines. Because we did not have information identifying individual respondents, and because some individuals were likely to have participated both in 2006 and 2009, we chose to use the hospital as the unit of analysis. The primary endpoint for statistical analysis was defined as the proportion of respondents in a particular hospitalwho always wash their hands before performing neuraxial block *and* always wear a facemask. For convenience, we called it "handwash-mask composite". Five secondary endpoints for statistical analysis were defined as the proportion of respondents who answered "always" in response to: 1) handwashing, 2) removal of watch or jewelry, 3) wearing face mask, 4) wearing sterile gown and 5) wearing hat/cap. For primary and secondary endpoints we compared "always" *vs* any other response ("usually, rarely or never").

We used logistic regression to perform the analysis. SAS Version *9.1* was used to analyze the data. Year (2006 and 2009) and hospital (1,2,3 or 4) were included in the analysis as fixed effects. For the single primary endpoint, we used a p-value of 0.05. For the five secondary endpoints, we used the Bonferroni-corrected family-wise error rate of 0.01 in order to control for multiple comparisons.

In order to identify individual-level determinants of hygiene compliance on our primary response variable "handwash-mask composite", we used a logistic regression model, stratified by year. We were not able to analyze both waves of the survey (Year 1 and Year 2) together because, while many of the respondents were the same in the two surveys, we were unable to match the respondents in the earlier and later waves of the survey. Consequently, we were unable to examine individual-level factors which may have been associated with changes in behavior.

The analysis for 2006 included the following variables: age, gender, professional status, years of experience, hospital, and number of deliveries per year in the responding hospital. The analysis for 2009 included all of those variables, as well as the variable representing the number of epidurals performed per month. Gender, position, and hospital were categorical variables and were considered as fixed effects. Age, years of experience, and numbers of deliveries were continuous variables.

## Results

The sample frame for the two surveys (2006 and 2009) was 160 and 164 anesthesiologists respectively. 135 and 127 anesthesiologists responded to the two surveys, representing a response rate of 84% and 77% respectively. The respondents constituted 23% of the national anesthesiologist workforce during each of the survey periods. Demographic data are presented in Table 1.

**Table 1:** Demographics of individual responding anesthesiologists and demographics of the participating hospitals

	**2006**	**2009**
***Demographics of participating anesthesiologists***		
	
Respondents/Frame (% response rate)	135/160 (84.4%)	127/164 (77%)
Age (years): Median (IQR)	46 (37-54)	42.5 (37-50)
Experience (years): Median (IQR)	14 (5-25)	10 (5-18)
Males	96/131	89/124
Females	35/131	35/124
Attendings	67/135	62/127
Residents	56/135	58/127
NRNA (non-resident, non-attending)	12/135	7/127
***Demographics of participating hospitals***		
Deliveries/year: Mean ± SD	8350 ± 2738	9222 ± 3253
% Epidural rate: Mean ± SD	45 ± 16	50 ± 18
% Cesarean rate: Mean ± SD	18 ± 5	18 ± 4

Continuous demographic variables were compared using one way ANOVA.

There were no significant differences between groups.

Note: Not all questions were answered by all respondents (95.5% of primary endpoint data in 2006, 99.5% in 2009), so the denominator is not constant throughout.

**Figure 1:**
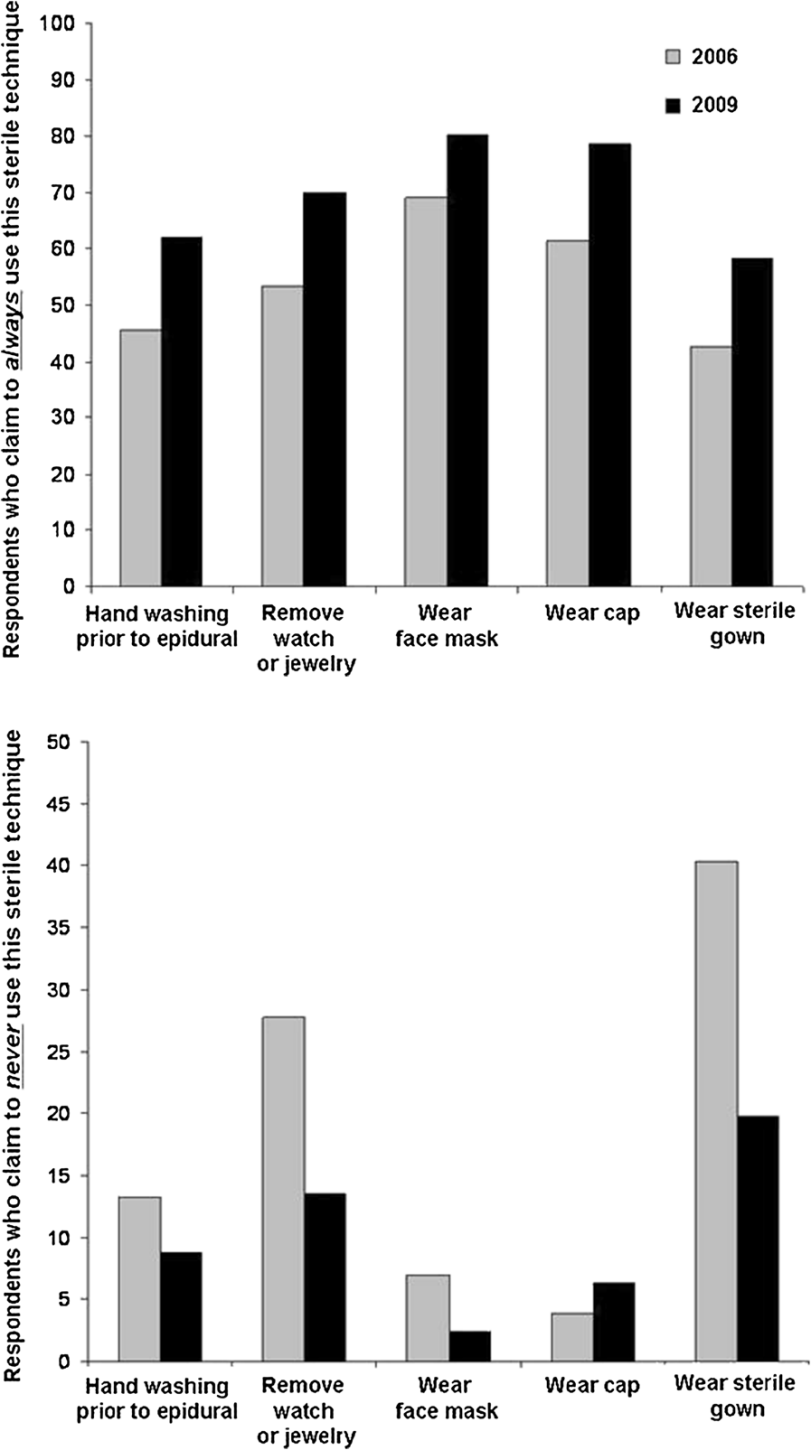
**Bar plot representing the percentage of responding anesthesiologists who either always (upper plot) or never (lower plot) adhere to the following aseptic techniques for neuraxial anesthesia: handwashing, removing jewelry or watches, wearing face mask, wearing a cap and wearing a sterile gown.** The data are compared for the year 2006 (prior to the ASRA guideline) and 2009.

A graph of the overall percentages of compliance before and after publishing the guidelines appears in Figure 1. Compliance was improved for all measures after publication of the guidelines. Averaged data for all the medical centers in the sampling frame are presented in Table 2 (raw data) and Table 3 (logistic regression data) and show that the main outcome "handwash-mask composite" increased after guideline publication (33% vs 58%; p = 0.0005). In addition, significant improvements were seen for handwashing alone, mask alone, gown and cap.

**Table 2:** Responders who "always" complied with specific elements of aseptic technique for neuraxial analgesia – raw descriptive data; 2006 and 2009

**Variable**	**Group**	**2006 Number/total**	**(%)**	**2009 Number/total**	**(%)**
Handwashing	Males	38/91	(41.8)	49/88	(55.7)
	Females	20/34	(58.8)	26/35	(74.3)
	Attendings	28/62	(45.2)	42/62	(67.7)
	Residents	29/55	(52.7)	34/58	(58.6)
	Non-residents	2/12	(16.7)	2/6	(33.3)
Removes jewelry watch	Males	49/88	(55.7)	68/88	(77.3)
	Females	15/34	(44.1)	18/35	(51.4)
	Attendings	30/62	(48.4)	43/62	(69.4)
	Residents	33/53	(62.3)	42/57	(73.7)
	Non-residents	4/11	(36.4)	3/7	(42.9)
Wears mask	Males	63/90	(70.0)	74/89	(83.1)
	Females	27/35	(77.1)	25/35	(71.4)
	Attendings	44/63	(69.8)	44/62	(71.0)
	Residents	42/54	(77.8)	54/58	(93.1)
	Non-residents	4/12	(33.3)	4/7	(57.1)
Wears sterile gown	Males	35/90	(38.9)	52/89	(58.4)
	Females	17/35	(48.6)	19/35	(54.3)
	Attendings	26/62	(41.9)	36/62	(51.6)
	Residents	26/55	(47.3)	37/58	(63.8)
	Non-residents	3/12	(25.0)	5/7	(71.4)
Wears cap	Males	58/91	(63.7)	72/89	(80.9)
	Females	22/35	(62.9)	25/35	(71.4)
	Attendings	37/63	(58.7)	43/62	(69.4)
	Residents	39/55	(70.9)	53/58	(91.4)
	Non-residents	4/12	(33.3)	4/7	(57.1)

Compliance with any specific aseptic technique defined as "always" versus all other responses ("usually, rarely or never").

Note: Not all questions were answered by all respondents (95.5% of primary endpoint data in 2006, 99.5% in 2007), so the denominator is not constant throughout.

**Table 3:** Responders who "always" complied with specific elements of aseptic technique for neuraxial analgesia – comparison between 2006 and 2009: percentages and results from logistic regression

**Variable**	**2006**	**2009**	**Effect of year (Odds ratio; 95% CI; p-value)**	**Effect of hospital (p-value)**
Handwash-mask composite (i.e., ALWAYS handwashing; ALWAYS mask)	**33%**	**58%**	0.32; 0.17–0.60; 0.0003	0.0005*
Handwashing	**37%**	**63%**	0.31; 0.17–0.59; 0.0003	0.0004**
Jewelry	**51%**	**69%**	0.48; 0.26–0.86; 0.014	0.26
Mask	**61%**	**78%**	0.29; 0.13–0.64; 0.0024	< 0.0001**
Gown	**32%**	**51%**	0.27; 0.13–0.55; 0.0004	< 0.0001**
Cap	**53%**	**76%**	0.31; 0.16–0.61; 0.0006	0.0011**

Compliance with any specific aseptic technique defined as "always" versus all other responses ("usually, rarely or never"). Percentages averaged from all hospitals in the sampling frame.

Statistical assessment based on logistic regression.

*Significant at the p < 0.05 level; (main outcome).

**Significant at the p < 0.01 corrected Bonferroni level.

In Table 4, we present the results of the logistic regression model, stratified by year, to assess the effect of specific demographic and other factors on our primary response variable of hygiene compliance, "handwash-mask composite". In 2006, there were 113 observations with complete data, of 135 total respondents. Men were significantly less likely to be fully compliant with aseptic technique (OR = 0.212, p < 0.01) than women. Residents were far more likely than NRNA's to be compliant (OR = 14.6, p < 0.01). No other variables were significantly associated with compliance. In 2009, there were 119 observations with complete data, of the total 127 observations. Younger age was associated with greater compliance (OR = 0.91, p = 0.05). As number of epidurals performed increased, compliance decreased (OR = 0.52, p = 0.01). No other variables were significantly associated with compliance. Hospital was considered a fixed variable, and was associated with significant impact on the increase in compliance with aseptic technique following the introduction of guidelines (p = 0.01); while the compliance increased in all hospitals to some degree, the OR for pre-post increase in guideline compliance for the different hospitals ranged from 1.04 (0.18-5.95) to 7.04 (1.30-38.19).

**Table 4:** Estimates of ORs (Odds Ratios) for the effects of demographic factors on compliance with aseptic technique for neuraxial analgesia (2006 and 2009 assessed separately)

**Effect**	**Point estimate**	**95% Wald confidence limits**	**p-values**
**2006**			
Age	0.95	0.85, 1.05	0.31
Male vs female*	0.21	0.07, 0.69	0.01
Resident vs NRNA*	14.62	1.94, 286.85	0.01
Attending vs NRNA	5.89	0.74, 64.58	0.60
Years experience	1.07	0.97, 1.17	0.18
No. deliveries/year	0.47	0.19, 1.14	0.10
**2009**			
Age*	0.91	0.83, 1.00	0.05
Male vs female	0.57	0.20, 1.59	0.28
Resident vs NRNA	0.81	0.07, 9.73	0.92
Attending vs NRNA	1.27	0.12, 12.98	0.69
Years experience	1.03	0.94, 1.14	0.52
No. deliveries/year	1.23	0.68, 2.21	0.49
No. epidurals/month*	0.52	0.31, 0.87	0.01

*Significant at the p < 0.05 level.

Compliance with any specific aseptic technique defined as "always" versus all other responses ("usually, rarely or never").

**Figure 2:**
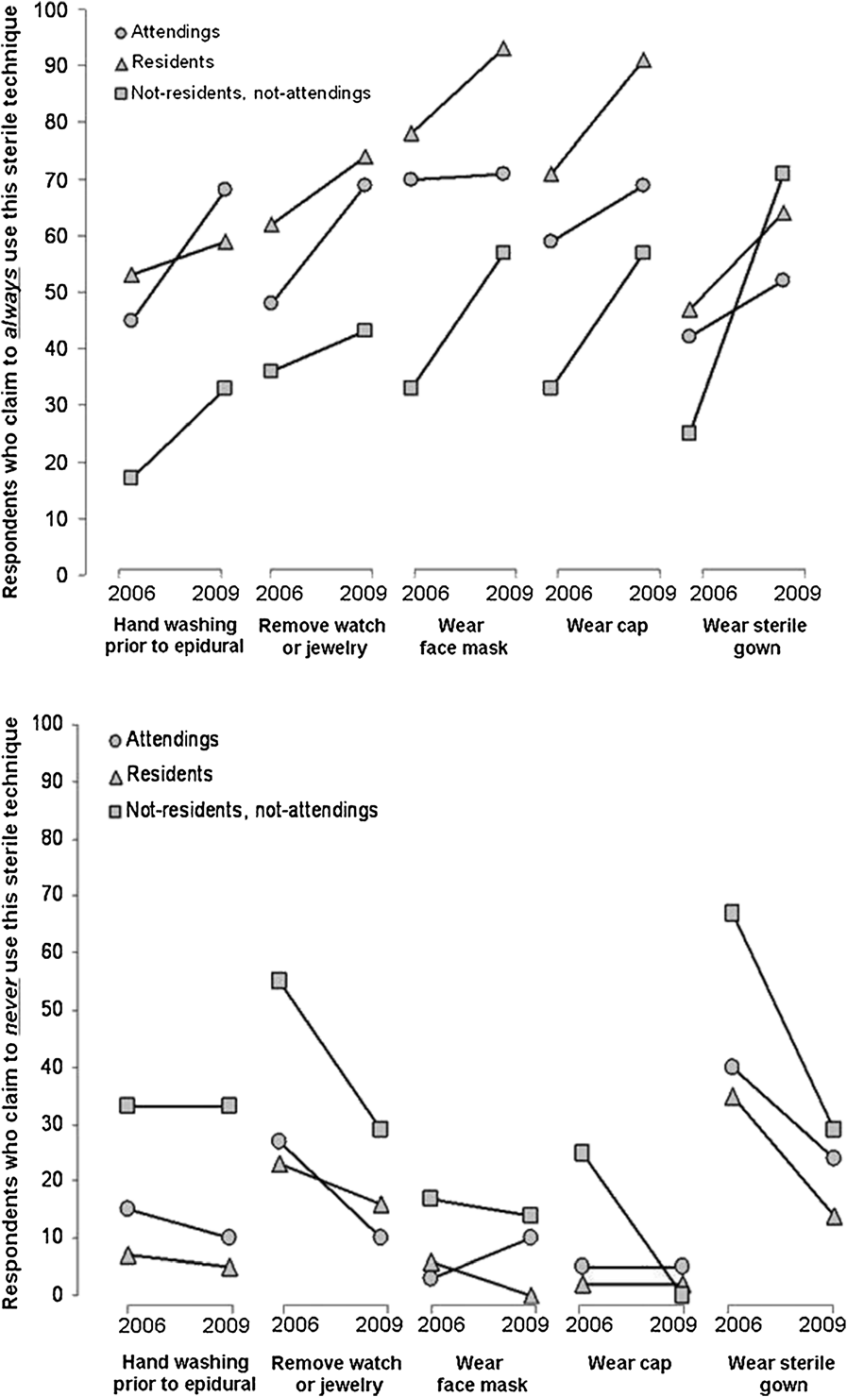
**Data for attendings, residents and non-residents, non-attendings (NRNA).** Paired line plot presenting the change from the baseline survey to the follow-up survey for each of the following aseptic techniques for neuraxial analgesia: handwashing, removing jewelry or watches, wearing face mask, wearing a cap and wearing a sterile gown. As for figure 1, the plot represents the percentage of responding anesthesiologists who either always (upper plot) or never (lower plot) adhere to the aseptic practices for neuraxial anesthesia. The data are compared for the year 2006 (prior to the ASRA guideline) and 2009. Attendings, residents and NRNA's (non-residents, non-attendings) are represented separately. See text for details of the inferential statistics.

Female anesthesthesiologists appear to be more compliant with handwashing than their male colleagues, but less inclined to remove their jewelry/watches (Table 2). The professional status of the anesthesiologist was a factor in the adherence to aseptic technique for handwashing, wearing a cap and wearing a mask. In these comparisons, the residents scored slightly better than the attendings and the NRNA's scored much worse (Table 2). All groups of physicians demonstrated an improvement in aseptic technique from 2006 to 2009 with an apparent narrowing of the difference in the second survey (Figure 2).

**Figure 3:**
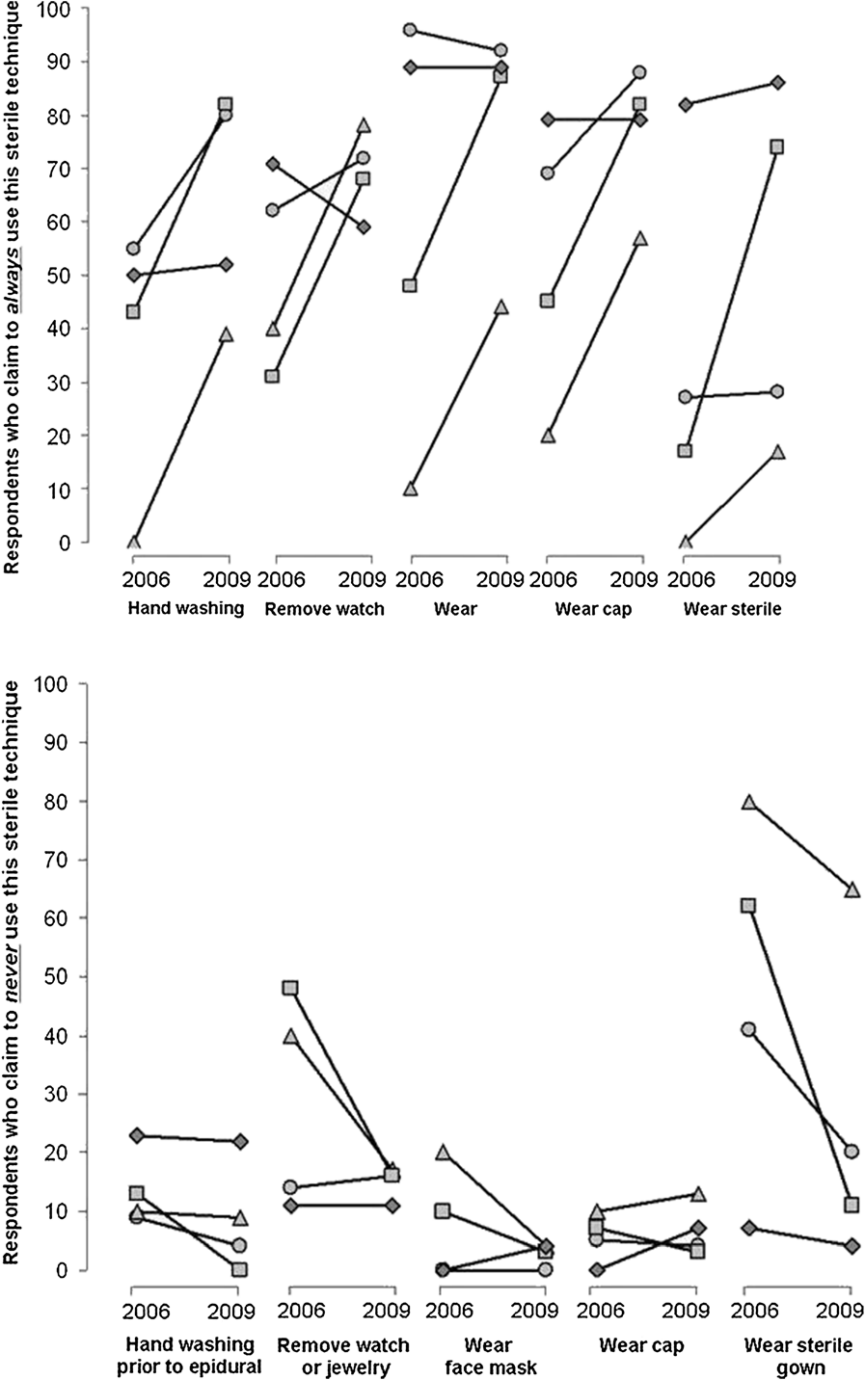
**Data for individual hospitals: Paired line plot presenting the change from the baseline survey to the follow-up survey for each of the following aseptic practices for neuraxial anesthesia: handwashing, removing jewelry or watches, wearing face mask, wearing a cap and wearing a sterile gown.** As for Figure 1, the plot represents the percentage of responding anesthesiologists who either always (upper plot) or never (lower plot) adhere to the aseptic practices for neuraxial anesthesia. The data are compared for the year 2006 (prior to the ASRA guideline) and 2009. Each symbol represents a specific hospital from each of the four Israeli medical schools; hospitals are not identified and data are not analyzed statistically. Nevertheless, two phenomena are apparent: 1) an improvement in aseptic practice from 2006 to 2009 in almost every hospital and in almost every aseptic practice assessed and 2) a wide inter-hospital variation in practice.

An apparent improvement is noted across all hospitals in almost all aseptic practices assessed (Figure 3). Of note is a marked inter-hospital variation in aseptic practice, both in the baseline aseptic technique and in the response to the guidelines between the two surveys.

Of the secondary endpoints, the most striking finding was an increase in the use of sterile disposable drapes from 11% to 62% from 2006 to 2009 (instead of sterile re-usable drapes). 46% of respondents wash with soap, 20% with alcohol-based antiseptic solution, 9% with both soap and alcohol-based antiseptic solution, 11% using surgeons' scrub protocol and 13% do not wash (prior to neuraxial analgesia). 98% prep the patient's back using chlorhexidine-alcohol, while 2% use povidine-iodine. 97% use clear sterile polyethelene dressing placed over the epidural site.

Thirty-seven percent of respondents claim that written guidelines exist in their institution for aseptic technique in neuraxial anesthesia (increased from 31% in 2006 to 42% in 2009); 33% claim that there are no such guidelines (reduced from 37% in 2006 to 29% in 2009) and 31% do not know (reduced from 33% in 2006 to 28% in 2009). Eighty percent consider that there is no difference in the performance of their aseptic technique in the labor ward to that performed by them in the OR. Eighty-five percent feel that their personal level of aseptic technique is adequate (8% better than adequate, 8% less than adequate). Seventy-nine percent feel that their personal level of aseptic technique is comparable to that of their colleagues (20% better than their colleagues and, interestingly, only 2% worse than their colleagues). In the 2006 survey, a striking 12% of respondents claimed to have had a major infectious complication (epidural abscess or meningitis) in a neuraxial block that they personally performed. This number was only 3% in 2009.

There was a clear disparity between the standards of precaution taken by the anesthesiologist and those expected of the midwife/nurse and the patient and family members present during block placement. Only 13% and 10% of respondents always ensure that the midwife and partner (respectively) wear a mask during the placement of the block, while 59% and 69% never do. Similarly, only 13%, 13% and 8% of respondents always make the midwife, patient and husband (respectively) wear a cap during the placement of the block, while 56%, 54% and 76% of respondents never do.

## Discussion

Monitoring the impact of an intervention is an important component in public health. In 2006, we felt that the impending ASRA guidelines on aseptic technique for neuraxial anesthesia should be regarded as a public health intervention. As in many other broad public health interventions, there was no control group available. Consequently, we took a before-and-after approach to trying to understand whether this intervention may have been beneficial. Although the before-after study design cannot prove a cause-effect relationship, it does show an association between the publication of international guidelines and significant improvement in self-reported aseptic technique.

We chose to conduct the follow-up survey at an interval of three years in order to avoid identifying short-term changes in aseptic technique that may have occurred following the guideline. The three year interval was sufficiently long for other interventions to have occurred during the same period. For example, the Israel Association of Obstetric Anesthesia held two national meetings devoted to infectious complications of neuraxial analgesia during the three year interval. Furthermore, there were several important publications in the anesthesia literature during the same period which focused on infectious complications following neuraxial anesthesia [1,8]. Finally, the Israel Society of Anesthesiologists (October 2009) and the American Society of Anesthesiologists (March 2010) [30] recently adopted a guideline and a practice advisory, respectively, for aseptic practices for neuraxial anesthesia. Although these guidelines were not published until after the completion of the second survey, the knowledge of their impending publication may also have had an impact on practice. Consequently, it is not possible to ascertain from these data whether the international guidelines were responsible for this change in self-reported aseptic technique practice.

It should be stated that the level of evidence for the efficacy of these sterile practices in preventing infectious complications of neuraxial analgesia is low (grade D) [30]. The choice of the handwashing-facemask composite was dictated by two facts: a) that poor handwashing routines constitute the most important preventable cause of nosocomial infection, and b) in the rare instances where infectious complications of neuraxial analgesia were attributed to bacterial contamination from the practitioner, these involved matching of pathogens in both the cerebrospinal fluid from the affected patients with an identical pathogen (e.g. *Streptococcus salivarius* identified by rDNA sequence analysis on polymerase chain reaction [4]), in the nasopharynx of the practitioner which may have been prevented by the use of a facemask.

This study shares the limitation of all practice surveys; responder bias and self-reporting. While the high response rates in this study limit the response bias, it is impossible to verify whether the self-reporting of increased compliance with aseptic technique actually reflects increased compliance in practice. The correlation between self-reported and observed adherence to handwashing guidelines has been reported to be poor [31]. Nevertheless, despite identical methodology in the two surveys, the results showed a marked change over the study period. Whether this is due to a genuine increase in adherence to aseptic practice or whether this is due to social desirability response bias, both options imply that there is, at the very least, a greater awareness of the correct practices over this time period.

Interestingly, NRNA’s (anesthesiologists who have not passed their board exams but who continue to practice anesthesia under supervision after the expiration of their residency period) reported far worse aseptic technique than either residents or attendings. The commitment of an individual physician to the health care organization has been shown to have an independent effect on familiarity with clinical guidelines [32], an essential pre-requisite for compliance. A recent study demonstrated the positive impact on handwashing hygiene of the behavior of senior clinicians as positive role models [33], and there is potentially a cumulative effect on practice once role models (both senior and junior) start to implement guidelines. Finally, the non-compliance of senior role models has led to the erosion of compliance among other physicians [34].

While the causes for this observed change in practice may have been multifactorial, the fact that a change was observed is an encouraging sign of the implementation of changes in health care policy. The implication for health care policy at a national and a hospital level of the change in aseptic practice for neuraxial anesthesia is that change in clinical practice requires multiple levels of effort. In this case, initial clinical evidence was followed by consensus-based international professional guidelines (ASRA and ASA); these were reinforced by national professional guidelines (Israel Society of Anesthesia), HMO-based clinical directives (Clalit) and national academic meetings. Although prospective studies observing actual compliance with guidelines in clinical practice and registries of infectious complications of neuraxial anesthesia may be the ideal measures of clinical change, on-going surveys of self-reported practice provide a simple and quantifiable endpoint.

Our data show two interesting phenomena; a general improvement in standards of self-reported aseptic practice in the three years since the ASRA guidelines coupled with a marked inter-hospital variation. This inter-hospital variation was both evident in the baseline practice of aseptic technique and in the impact of the intervention. While national and international guidelines may have exerted a significant impact on the practice of aseptic technique by anesthesiologists across the spectrum of different hospitals, it is the translation of these guidelines into practice at local individual hospitals that will lead to uniform improvements in the standard of care.

These guidelines represent a broad consensus of acceptable practice. It is therefore of some concern that compliance is so far from universal. Two questions may be asked; why is compliance with guidelines disappointingly low and what can be done to improve it? These questions are not restricted either to hand washing, to the profession of anesthesiology or to the practice of medicine in Israel; they are ubiquitous throughout modern medicine.

Grol et al identified multiple causes for poor implementation of hand hygiene guidelines and stratified these by the health system strata where the lapse occurs [27]. Among individual practitioners, they identified problems as cognitive (unconvinced by the evidence), motivational (fear of hand irritation) and due to working routines. At the level of the health team (in our case, the anesthesia department), the problem was primarily due to lack of leadership, with poor control or accountability. At the level of the hospital, the problems were related to excessive workload demands, poor access to facilities and the lack of institutional policies. In a survey of doctors, residents and medical students, Erasmus et al identified the most important factors affecting adherence with handwashing hygeine guidelines to be the strength of evidence and the behavior of role-model mentors [35]. Pittet has applied social and cognitive models derived from the behavioral sciences in order to better understand the poor compliance of healthcare workers with hand washing hygiene guidelines [36,37].

Within the profession, the most obvious steps to be taken are at the departmental level: clear leadership and positive role models, backed up by focused educational programs, audit and feedback [30]. More difficult to assess is the degree to which coercive steps should be taken if voluntary compliance remains low. As guidelines specify how health care should be performed, they have “shifted the focus of professional power from autonomy to accountability” [38]. Many tools are available to local and national regulators and third party payers to monitor and even enforce compliance with practice guidelines, including disciplinary action, financial incentives based on healthcare quality indicators, or the publication of performance data [39]. However, it is probably more effective and certainly less intrusive for the profession to treat itself from within.

## Conclusion

Self-reported aseptic technique by Israeli anesthesiologists improved in the survey conducted after the publication of international guidelines. Although the before-after study design cannot prove a cause-effect relationship, it does show an association between the publication of international guidelines and significant improvement in self-reported aseptic technique.

## Appendix

American Society of Regional Anesthesia (ASRA): Practice Advisory (2006) [20-22].

1. Thorough hand washing greatly reduces the risk of cross-contamination and should occur before performing any regional anesthetic technique. Alcohol-based antiseptic solutions will provide the maximal degree of antimicrobial activity with extended duration when compared with nonalcoholic antimicrobial or nonantimicrobial preparations (Grade A). The duration and method of washing (standard hand washing vs. full surgical scrub) required to reduce infectious complications is currently unknown.

2. Higher microbial counts have been identified in health care workers who do not remove jewelry before hand washing. Therefore, it may be prudent to remove all jewelry items (rings, watches, and so on) before hand washing to reduce the risk of contamination (Grade B).

3. Sterile surgical gloves should be used and considered a supplement to, not replacement for, hand washing. The use of surgical gloves is ad-vocated not only to protect patients from cross-contamination but also to protect health care workers from blood-borne pathogen exposure as required by the Occupational Safety and Health Administration (Grade A).

4. Several intensive care unit–based investigations have shown that the use of surgical gowns does not reduce patient colonization, infection, or mortality rates beyond that achieved with gloves alone. However, there is currently insufficient data to make recommendations with regard to routine use during regional techniques within the operating room environment.

5. The use of surgical masks during regional anesthesia will maximize sterile barrier precautions. In particular, surgical masks have been found to significantly reduce the likelihood of site contamination from microorganisms grown in the upper airway of clinicians. Although the routine use of masks have not been found to reduce infectious complications related to regional anesthesia, they do remain a vital protective measure against bloodborne pathogen exposure as recommended by the Occupational Safety and Health Administration (Grade B).

6. Currently, the literature does not support the routine use of bacterial filters with short-term (i.e., days) epidural or perineural catheter infusions (Grade B).

7. Alcohol-based chlorhexidine antiseptic solutions significantly reduce the likelihood of catheter and site colonization and maximize the rapidity and potency of bactericidal activity when compared to other solutions. Therefore, alcohol-based chlorhexidine solutions should be considered the antiseptic of choice before regional anesthetic techniques (Grade A).

Israel Society of Anesthesiologists (ISA) Practice Guidelines (2009).

Epidural or spinal anesthesia or analgesia should be performed under sterile conditions, including: handwashing with septol, use of sterile gloves, cap, mask and sterile gown. The assistant (eg midwife) should wear a cap and mask.

American Society of Anesthesiologists (ASA): Practice Advisory (2010) [26].

Aseptic techniques should always be used during the preparation of equipment (e.g., ultrasound) and the placement of neuraxial needles and catheters, including the following:

Removal of jewelry (e.g., rings and watches), hand washing, and wearing of caps, masks (covering both mouth and nose and consider changing before each new case), and sterile gloves.

Use of individual packets of antiseptics for skin pre-paration.

Use of chlorhexidine (preferably with alcohol) for skin preparation, allowing for adequate drying time.

Sterile draping of the patient.

Use of sterile occlusive dressings at the catheter insertion site.

## Competing interests

The authors declare that they have no competing interests.

## Authors’ contributions

AI designed the study, wrote the questionairre,supervised data collection in the different hospitals and helped write the manuscript. EMD provided valuable clinical insights, helped design the questionairre and helped write the manuscript. SOZ helped supervise data collection. ZR helped supervise data collection. SI helped supervise data collection. LJR performed the statistical analysis. AA designed the questionairre and analyzed the questionairre data. YG designed the study, wrote the questionairre, helped supervise data collection and wrote the manuscript. All authors read and approved the final manuscript.
